# Overexpression of SRC‐3 promotes esophageal squamous cell carcinoma aggressiveness by enhancing cell growth and invasiveness

**DOI:** 10.1002/cam4.884

**Published:** 2016-10-26

**Authors:** Fang‐Ping Xu, Yan‐Hui Liu, Xin‐Lan Luo, Fen Zhang, Hai‐Yu Zhou, Yan Ge, Chao Liu, Jie Chen, Dong‐Lan Luo, Li‐Xu Yan, Ping Mei, Jie Xu, Heng‐Guo Zhuang

**Affiliations:** ^1^ Department of Pathology and Laboratory MedicineGuangdong General Hospital & Guangdong Academy of Medical ScienceGuangzhouChina; ^2^ Department of Thoracic SurgeryCancer CenterGuangdong General HospitalGuangzhouChina

**Keywords:** Cell growth, cell invasion, esophageal squamous cell carcinoma, insulin‐like growth factor/AKT, steroid receptor coactivator‐3

## Abstract

Steroid receptor coactivator‐3 (SRC‐3), a transcriptional coactivator for nuclear receptors and other transcription factors, plays an important role in the genesis and progression of several cancers. However, studies investigated the role of SRC‐3 in esophageal squamous cell carcinomas (ESCCs) are limited, and the role of SRC‐3 in tumor progression remains unclear. We examined the expression of SRC‐3 in 8 ESCC cell lines and 302 human ESCC tissues by qPCR, Western blot, and immunohistochemistry. In addition, ESCC cell lines were subjected to proliferation and invasion assays, tumorigenicity assay, flow cytometry assay, qPCR, Western blot, and Chromatin Immunoprecipitation assay to investigate the role of SRC‐3 in cancer progression. SRC‐3 was overexpressed in 48% of cases and correlated with poor overall (*P* = 0.0076) and progression‐free (*P* = 0.0069) survival of surgically resected ESCC patient. Cox regression analysis revealed that SRC‐3 is an independent prognostic marker. Furthermore, we found that activation of insulin‐like growth factor (IGF)/AKT) was involved in the SRC‐3 on the cell growth and invasiveness in two ESCC cell lines, Eca109 and EC18 cells. SRC‐3 overexpression is clinically and functionally relevant to the progression of human ESCC, and might be a useful molecular target for ESCC prognosis and treatment.

## Introduction

Esophageal cancer is the sixth most common cause of death and eighth most common cancer in the world 1. Esophageal squamous cell carcinoma (ESCC) accounts for more than 90% of the esophageal cancer cases and is predominant in East Asian countries, especially in China 2. Despite the development of multimodality therapies, the prognosis of patients remains poor, even for those who undergo complete resection of their carcinomas 3, 4. Therefore, there is a great need to discover more biomarkers and therapeutic targets for ESCC patients.

Steroid receptor coactivator‐3 (SRC‐3; AIB1/ACTR/RAC3/p/CIP/NCoA‐3) is a member of the p160 SRC family. SRC‐3 has histone acetyltransferase activity and interacts with multiple nuclear receptors and transcription factors to regulate the expression of their target genes, which has been reported to be involved in a number of biological processes, such as cell proliferation, differentiation, migration, and others 5, 6. SRC‐3 is well studied in breast cancer and prostate cancer. Overexpression of SRC‐3 has been demonstrated to associate with poor disease‐free survival and play an important role in the genesis and progression of some breast cancers and prostate cancers 7, 8. Elevated SRC‐3 gene and protein expression are also found in many other hormone‐independent cancers, including human gastric, pancreatic, bladder, liver, and lung cancers, and correlated with cancer cell proliferation, invasiveness, and poor prognosis of the tumor 9, 10, 11, 12, 13, 14. Knockdown of SRC‐3 in lung cancers not only reduces cell growth and proliferation of non–small cell lung cancer cell lines but also potentiates the effects of gefitinib in EGFR tyrosine kinase inhibitor‐resistant cells 14. Those studies indicate that SRC‐3 plays an oncogenic role in carcinomas. Our previous study indicated that SRC‐3 was amplified and overexpressed in a subset of ESCCs, suggesting a potential impact of SRC‐3 on ESCC 15. However, studies investigating the role of SRC‐3 in ESCC are limited, and the knowledge of the function of SRC‐3 in ESCC cell growth and invasiveness is also to be revealed.

Herein, we show that overepression of SRC‐3 correlated with poor progression‐free and overall survival of surgically resected ESCC patient. Downregulation of SRC‐3 decreased the aggressive phenotype of ESCC cells both in vitro and vivo. Furthermore, our studies supported that the oncogenic effects of SRC‐3 might be through enhancing insulin‐like growth factor (IGF)/AKT pathway.

## Materials and Methods

### Case selection and tissue microarray construction

In this study, the paraffin‐embedded pathological specimens from 315 patients with ESCC were obtained from the Guangdong General Hospital (Guangzhou, China) between July 2004 and July 2009. None of the patients received neoadjuvant therapy before surgery. Clinicopathologic characteristics were detailed in Table 1. The tissue microarray (TMA) was constructed according to a method described previously 15. Briefly, H&E stained slides from each tumor block were used as guide to identify areas representing different stages of progression from each case, which were sampled using a tissue arraying instrument (Beecher Instruments, Silver Spring, MD) to remove a 0.6‐mm‐diameter cylinder of tissue. Then, the cylinder was reembedded into a predetermined position in a recipient paraffin block. In this ESCC TMA, two cores of sample were selected from each tumor and one core from adjacent nonneoplastic esophageal mucosa of the same patients. Tumor grade and stage were defined according to the criteria of the WHO (2010) and the 7th edition of the TNM classification of the AJCC (2010). The Institute Research Medical Committee of Guangdong General Hospital gave approval for this study.

**Table 1:** Correlation between the clinicopathologic features and Steroid receptor coactivator‐3 expression in ESCCs

Characteristic	Cases	Overexpression (%)	*P* value^a^
Age			0.277
≤58 yrs	160	81 (50.6%)	
>58 yrs	142	63 (44.4%)	
Gender			0.627
Male	229	111 (48.5%)	
Female	73	33 (45.2%)	
Histologic differentiation			0.200
Well	47	25 (53.2%)	
Moderate	201	99 (49.3%)	
Poor	54	20 (37.0%)	
T Classification			**0.020**
T1‐2	115	45 (39.1%)	
T3‐4	187	99 (52.9%)	
N classification			0.726
N0	150	70 (46.7%)	
N1‐3	152	74 (48.7%)	
M classification			0.909
M0	296	141 (47.6%)	
M1	6	3 (47.7%)	
Clinical stage			0.062
I–II	172	74 (43.0%)	
III–IV	130	70 (53.8%)	

a Chi‐Square test. Bold value here highlight the significant value (*P* <0.05)

### Immunohistochemistry (IHC)

See Appendix S1.

### Cell culture

Eight human ESCC cell lines, including CaES‐17, EC18 (gift from Professor Guan), Eca109, EC9706, KYSE‐140, KYSE‐450,KYSE‐510, and TE‐1, one immortalized human normal esophageal epithelial cell (HEEC), and one human embryonic kidney 293 (HEK293) cell lines purchased commercially were maintained in Dulbecco's modified Eagle's medium (Invitrogen, San Diego, CA) supplemented with 10% fetal bovine serum (HyClone, Logan, UT) and 1% penicillin/streptomycin in a humidified atmosphere with 5% CO_2_. All of the cell lines have been DNA fingerprinted for provenance using the Goldeneye^™^20A STR Multi‐amplification Kit. The DNA fingerprint was all confirmed to the same as in the DNA fingerprint library maintained by ATCC, DSMZ, JCRB, or the primary source of the lines. The lines were also tested to be free of mycoplasma contamination.

### Vectors, retroviral infection, and stable cell line selection

See Appendix S1.

### RNA extraction, cDNA synthesis, and qPCR

See Appendix S1.

### Western blot analysis

#### 5‐bromo‐2′‐deoxyuridine (BrdU) incorporation assays and Methyl thiazolyl tetrazolium (MTT) assays

See Appendix S1.

#### Colony formation and soft argar assays

See Appendix S1.

#### Flow cytometry assay

See Appendix S1.

#### Wound healing assay and transwell assay

See Appendix S1.

#### Chromatin immunoprecipitation (ChIP) assay

See Appendix S1.

#### Tumorigenicity assay

The study protocol was approved by and performed in accordance with the guidelines of the laboratory animal ethics committee of Guangdong General Hospital. For SRC‐3 transplantation studies, retroviral‐delivered shRNA SRC‐3 and shRNA scramble control cells were injected subcutaneously into the flank of 4‐week‐old male athymic nude mice (five mice per group; BiKai Co., Shanghai, China) in complete medium. Tumor diameters were measured with digital calipers, and the tumor volume was calculated in cubic millimeters. Animals were followed for 32 days, and all animals were sacrificed and the tumor weights were measured at the conclusion of the experiment.

### Statistical analysis

Statistical tests for data analysis included Fisher exact test or Chi‐square test, log‐rank test, and Student two‐tailed t‐test. Survival curves were assessed by the Kaplan–Meier method and compared by the log‐rank test. Relative risks of cancer‐related death associated with SRC‐3 expression status and other predictor variables were estimated by univariate analyses. Multivariate survival analysis was done on all parameters that were found to be significant on univariate level using the Cox regression model. Covariates including patient's age, gender, tumor grade, SRC‐3 expression, TNM, and clinical stage were used to adjust for in the Cox proportional hazard model. Statistical analyses were performed using the SPSS 13.0 statistical software package (SPSS Inc, Chicago, IL). A *P* value of less than .05 was considered significant.

## Results

### SRC‐3 overexpression correlates with progression and poor prognosis in human ESCC

**Figure 1:**
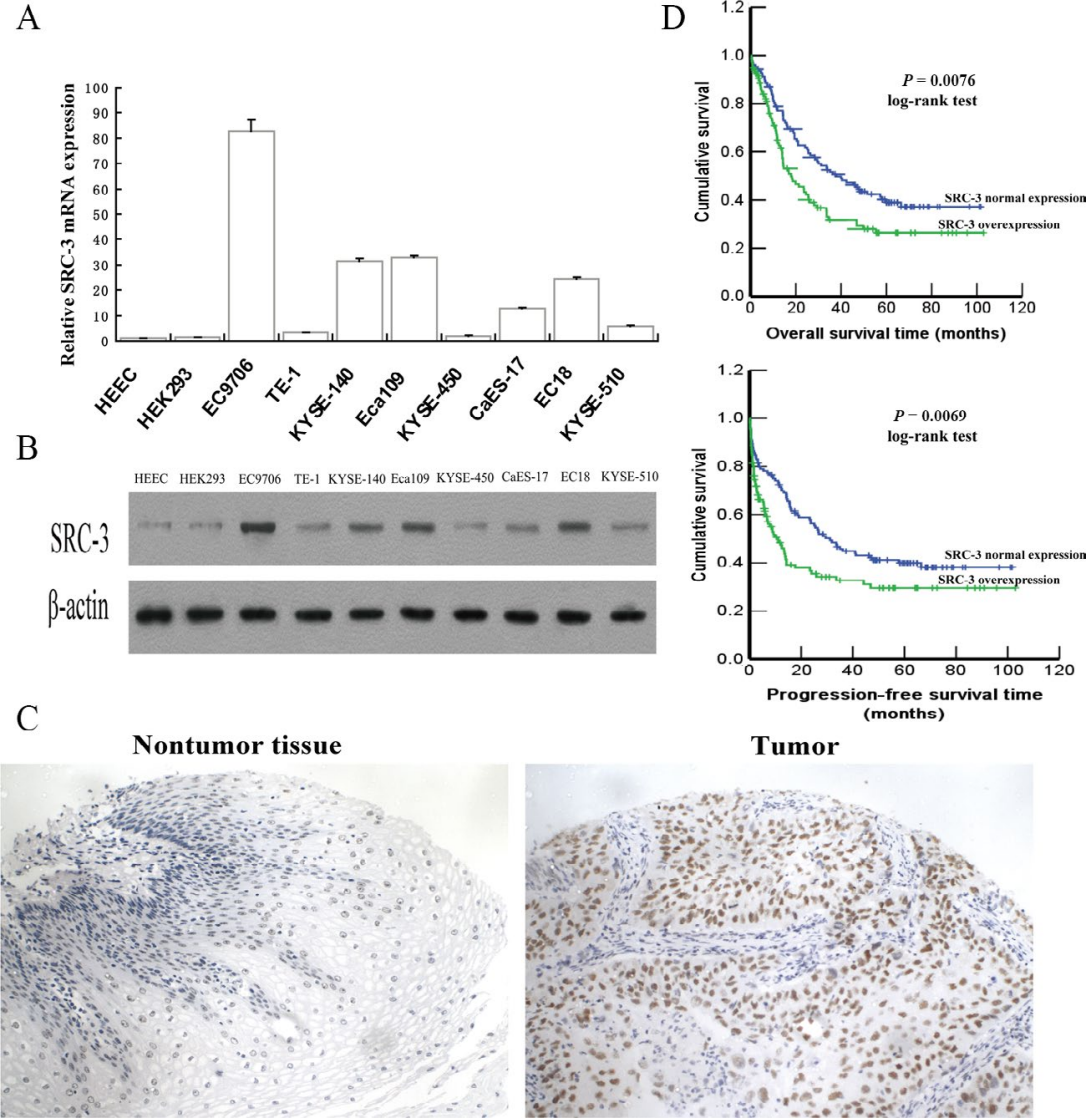
Steroid receptor coactivator (SRC)‐3 is upregulated in esophageal squamous cell carcinomas (ESCC) cell lines and primary human ESCC. (A and B) SRC‐3 mRNA and protein expression level in a panel of cultured ESCC lines, one HEEC and one HEK293 cell lines, by qPCR (A) and Western blotting analysis (B). (C) Representative immunohistochemical images showing SRC‐3 expression are upregulated in human ESCC compared with normal esophageal tissue. (D) Kaplan–Meier curves of ESCC patients with normal versus overexpression of SRC‐3.

To investigate the oncogenic role of SRC‐3 in ESCC progression, we first examined the expression of SRC‐3 in ESCC cell lines and human ESCC tissues. As shown in Figure 1A and B, SRC‐3 was upregulated at both the protein and messenger RNA (mRNA) levels in all eight analyzed ESCC cell lines compared to one primary HEEC and one HEK293 cells. The expression level of SRC‐3 was examined in 315 paraffin‐embedded, archived ESCC tissues using IHC. Informative expression of SRC‐3 was detected in 302 ESCC cases. SRC‐3 was markedly upregulated in ESCC but was only detectable at low levels in normal esophageal tissues (Fig. 1C). The results of the IHC analysis are summarized in Table 1. Overexpression of SRC‐3 was detected in 144 of 302 (47.7%) of informative ESCC cases. Statistical analyses revealed that SRC‐3 expression was significantly associated with advanced tumor stage (*P *=* *0.020) and tended to be more frequent in clinical stage III–IV (*P *=* *0.062) patients with ESCC. Therefore, upregulation of SRC‐3 in ESCC specimens as well as ESCC cell lines suggests that SRC‐3 may be involved in ESCC progression.

Moreover, Kaplan–Meier analysis was performed on the 242 patients subset for which survival data were available. As shown in Figure 1D, patients whose tumors have high SRC‐3 levels by immunohistochemistry have significantly shorter overall (*P* = 0.0076, log‐rank test) and progression‐free (*P* = 0.0069, log‐rank test) survival time than patients with low SRC‐3 levels. The mean overall survival time was 39.0 months for the SRC‐3 overexpression group and 52.3 months for the SRC‐3 normal expression group. By univariate analysis, overexpression of SRC‐3 (*P* = 0.006), advanced tumor stage (*P* < 0.0001)), presence of lymph node metastasis (*P* < 0.0001), and advanced clinical stage (*P* < 0.0001) were significant negative prognostic factors for OS in ESCC patients (Table 2). In the multivariate analysis, overexpression of SRC‐3 (*P* = 0.007) and advanced tumor stage (*P* = 0.017) retained independent significant predictive value for survival enrolled in this study (Table 3).

**Table 2:** Results of Univariate Cox Proportional Hazards Regression Analysis

Variable	No. of cases	*P*	RR	95% ConfidenceInterval for RR	
				LowerBound	UpperBound
Age (≤58 vs. >58)	116/126	0.231	1.223	0.880	1.701
Gender (Female vs. Male)	58/184	0.741	1.065	0.732	1.550
Differentiation (Well/Moderate vs. Poor)	41/201	0.516	1.149	0.756	1.748
SRC‐3 (Overexpression vs. Normal)	118/124	0.006	1.558	1.140	2.211
T classification (T3‐4 vs. T1‐2)	155/87	0.000	1.906	1.342	2.716
N classification (N1‐3 vs. N0)	126/116	0.000	1.874	1.333	2.634
M classification (M1 vs. M0)	4/238	0.070	3.668	0.898	14.979
Clinical stage (III‐IV vs.I‐II)	105/137	0.000	2.013	1.442	2.809

RR, relative risk; SRC, Steroid receptor coactivator.

**Table 3:** Results of Multivariate Cox Proportional Hazards Regression Analysis

Variable	No. ofcases	*P*	RR	95% ConfidenceInterval for RR	
				LowerBound	UpperBound
SRC‐3 (Overexpression vs. Normal)	118/124	0.007	1.582	1.134	2.207
T classification (T3‐4 vs. T1‐2)	155/87	0.017	1.657	1.092	2.512

RR, relative risk; SRC, Steroid receptor coactivator.

### Downregulation of SRC‐3 inhibits ESCC cell proliferation

**Figure 2:**
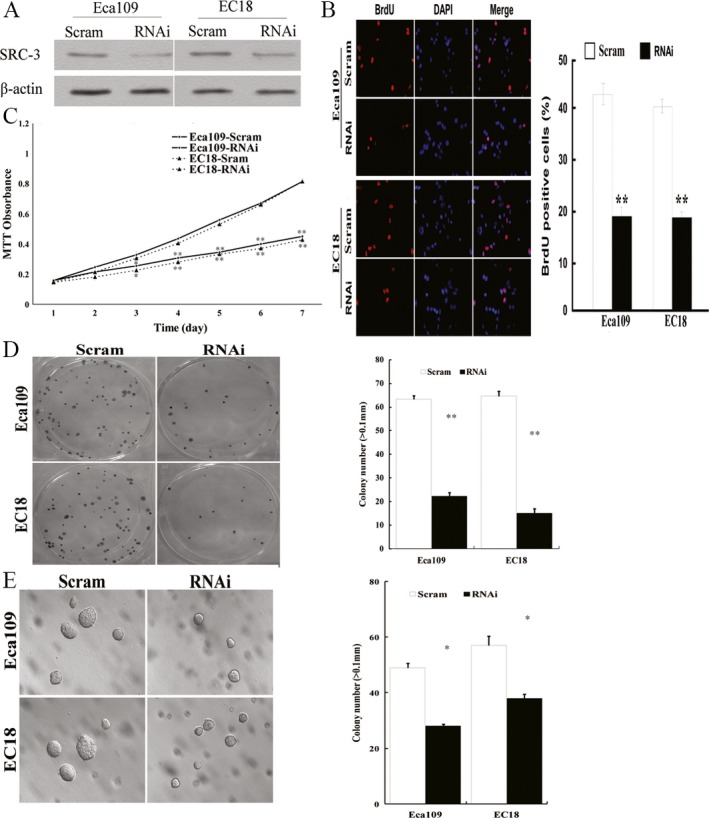
Knockdown of steroid receptor coactivator (SRC)‐3 in esophageal squamous cell carcinomas (ESCC) cell lines decreases cell growth. (A) SRC‐3 is efficiently depleted in specific short hairpin RNA‐transduced stable ESCC cell lines. *β*‐actin was used as a loading control. Scram, Scramble control; RNAi, SRC‐3–specific shRNA. (B) The representative pictures (left panel) and quantification (right panel) of cell proliferation rate of indicated cells as determined by BrdU incorporation. (C) Growth curves of indicated cell by MTT assay. (D) The representative pictures (left panel) and quantification (right panel) of crystal violet stained indicated cells. (E) The representative pictures (left panel) and quantification (right panel) of colony numbers of indicated cells as determined by an anchorage‐independent growth assay. Colonies larger than 0.1 mm in diameter were scored. *P <* *0.05 and **P <* *0.01.

In order to evaluate the role of SRC‐3 in ESCC cell function, two ESCC cell lines, Eca109 and EC18, expressed a moderate level of SRC‐3 were selected to generated stable SRC‐3 knockdown ESCC cell lines using shRNA transfection. Western blot experiment confirmed significant reduction in SRC‐3 expression in SRC‐3 knockdown cells relative to a scrambled control shRNA (Fig. 2A). As shown in Fig. 2B, the proliferation rate of endogenous SRC‐3 knockdown Eca109 cells, as assessed by the BrdU assay, was significantly reduced to 44% compared to the scrambled control cells. A similar result was found in EC18 cells. In the MTT assay, relative cell viability of SRC‐3 knockdown Eca109 and EC18 cells was significantly reduced to 55% and 52% by the seventh day, respectively (Fig. 2C).

To further characterize the effects of SRC‐3 on tumor cell proliferation, a colony formation assay was performed. As shown in Figure 2D and E, silencing SRC‐3 significantly decreased the colony formation in both assays, demonstrating that SRC‐3 enhances anchorage‐independent growth and colony formation.

### Downregulation of SRC‐3 arrests ESCC cell cycle at G1/S transition

**Figure 3:**
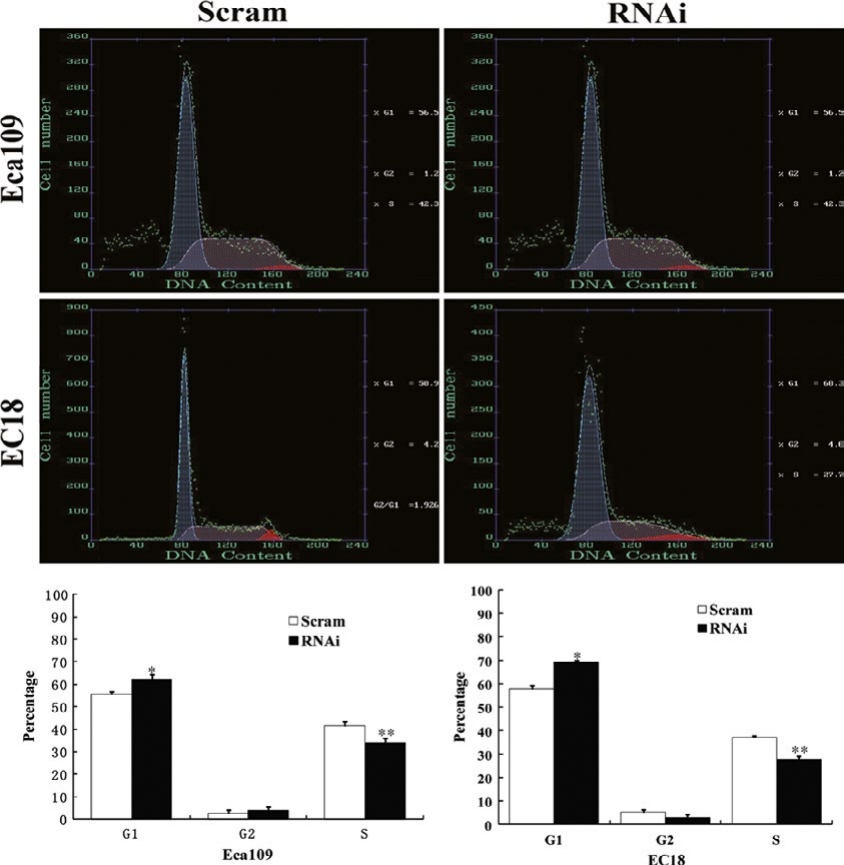
Downregulation of SRC‐3 inhibits ESCC cell cycle progression. The representative pictures (upper panel) and quantification (under panel) of cell cycle of indicated cells performed by flow cytometry. *P <* *0.05 and **P <* *0.01. ESCC, esophageal squamous cell carcinomas. SRC, Steroid receptor coactivator.

To elucidate the mechanism underlying growth inhibition by downregulation of SRC‐3, flow cytometric analysis was performed to compare cell distributions in cell cycle between SRC‐3 knockdown cells and control cells. The percentage of SRC‐3 knockdown cells in G0/G1 phases was increased by 11% and 20% in Eca109 and EC18 cells, respectively; and this was associated with a concomitant decrease in cell in S phase compared with that in control cells, suggesting that SRC‐3 knockdown was able to inhibit DNA synthesis and arrest cell cycle at G1/S transition (Fig. 3).

### SRC‐3 enhances ESCC cell migration and invasion

**Figure 4:**
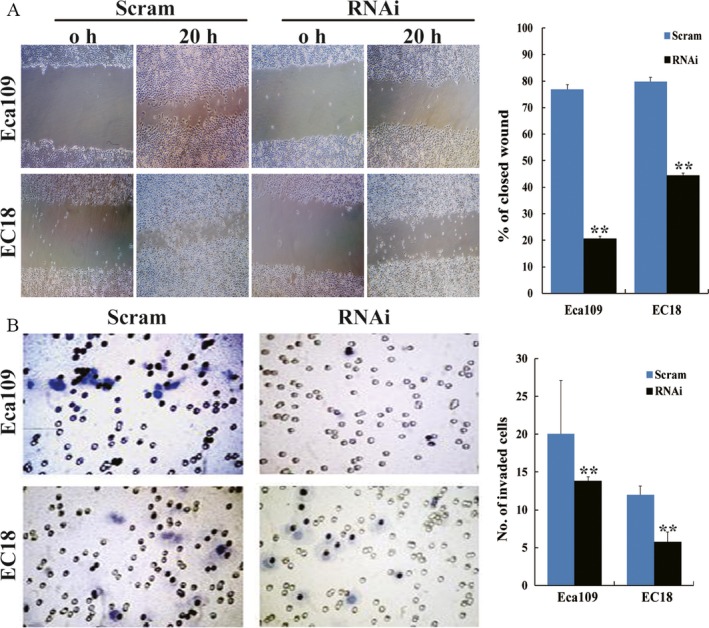
SRC‐3 enhances ESCC cell migration and invasion. (A) The representative pictures (left panel) and quantification (right panel) of indicated cells migrated into a defined wound area after 20 h. (B) The representative pictures (left panel) and quantification (right panel) of invaded cells were analyzed using the Transwell matrix penetration assay. **P <* *0.01. ESCC, esophageal squamous cell carcinomas; SRC, Steroid receptor coactivator.

As the overexpression of SRC‐3 examined by immunohistochemistry was positively associated with ESCC advanced tumor stage and ascending clinical stage, the effects of SRC‐3 on ESCC cell migration and invasion were studied by wound healing and cell invasion assays, respectively. Wound healing assay showed that, 20 h after a wound was made on the monolayer of cells, the spreading speed of SRC‐3 knockdown cells along the wound edge was slower than that in control cells, demonstrating that depletion of endogenous SRC‐3 could dramatically inhibit cell migration ability in both Eca109 and EC18 cells (Fig. 4A). Matrigel invasion assay also found that knockdown of SRC‐3 could inhibit the invasiveness of ESCC cells, as showed by a significant decrease in the number of invaded cells in Eca109 and EC18 cells compared to scramble control (*P* < 0.001, Fig. 4B). These results suggest that SRC‐3 plays a critical role in migration and invasion of human ESCC cells.

### SRC‐3 contributes to the progression of ESCC in vivo

**Figure 5:**
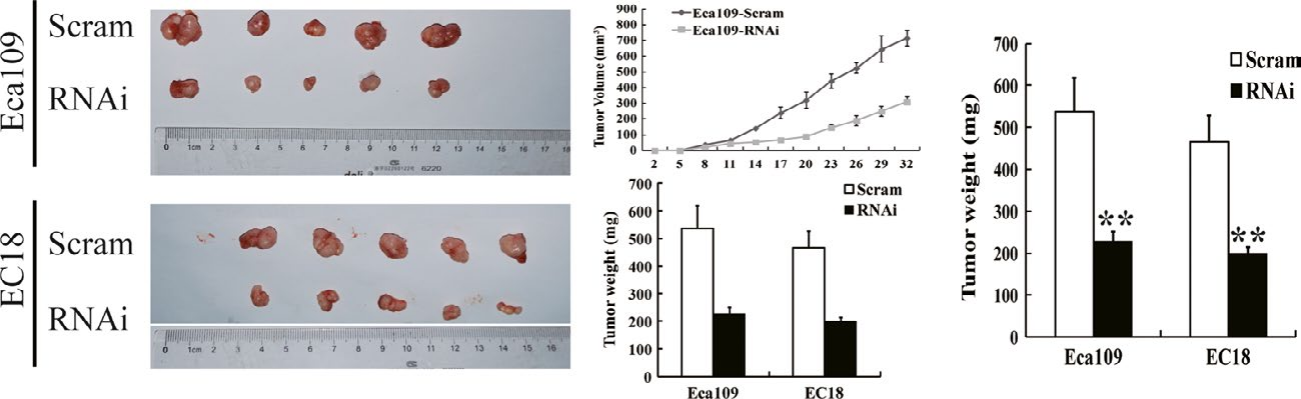
SRC‐3 contributes to tumorigenesis as examined in a xenograft model. Images of the tumors from all mice in each group (left panel). Tumor volumes were measured on the indicated days (middle panel). The tumor masses were determined with the scale (right panel). **P <* *0.01. SRC, Steroid receptor coactivator.

To further investigate the role of SRC‐3 in ESCC progression, we assessed the effects of SRC‐3 knockdown on the growth of ESCC xenograft tumors in nude mice injected with SRC‐3 knockdown or control cells. SRC‐3 knockdown Eca109 and EC18 tumors grew much slower than control tumors. At the end of the study (day 32), tumor size of SRC‐3 knockdown group (309 ± 34 mm^3^, 293 ± 27 mm^3^) was only 43% and 48% of the control group (715 ± 51 mm^3^, 616 ± 27 mm^3^), respectively, and tumor weight of SRC‐3‐silenced group (229 ± 22 mg, 201 ± 14 mg) was also significantly lower than the scramble control group (537 ± 82 mg, 466 ± 61 mg), respectively (Fig. 5). These results indicate that SRC‐3 contributes to the progression of ESCC cells in vivo.

### SRC‐3 activates Insulin‐like growth factor/AKT signaling pathway

**Figure 6:**
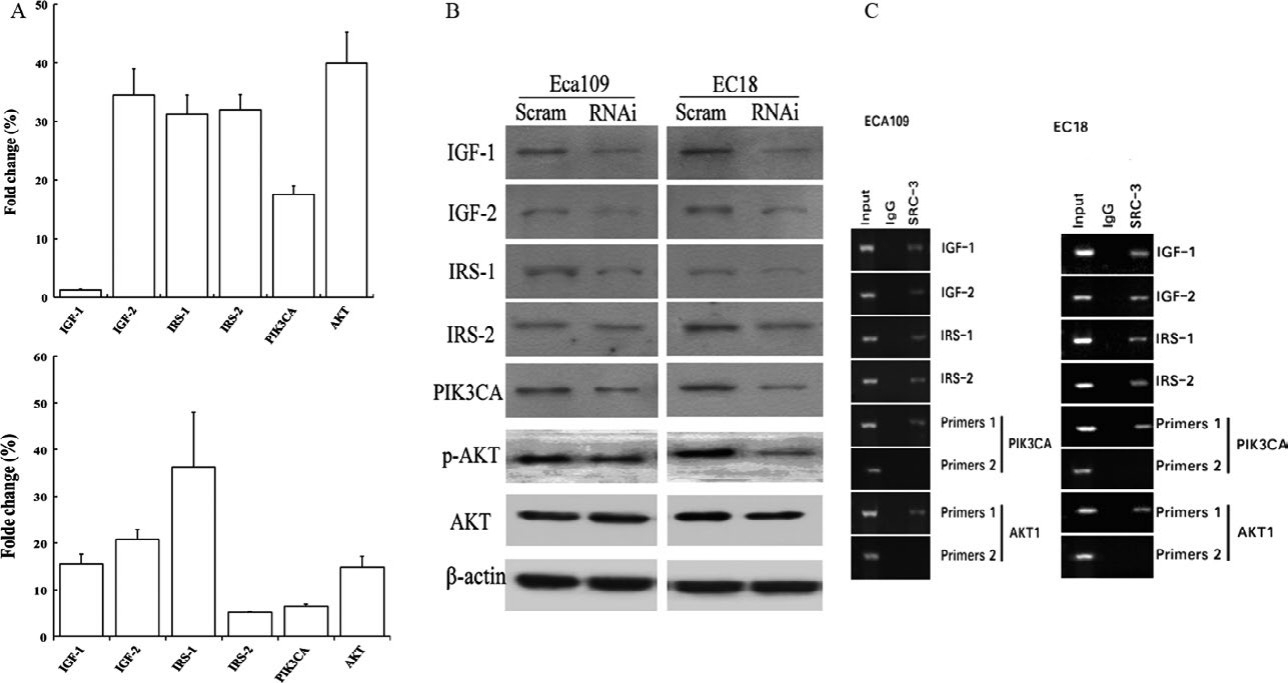
Steroid receptor coactivator (SRC)‐3 activates insulin‐like growth factor (IGF)/AKT signaling. (A) Fold changes of mRNA expression level of IGF/AKT components, including IGF‐I, IGF‐II, IRS‐1, IRS‐2, PIK3CA, AKT from indicated cells by qPCR in SRC‐3 knockdown cells compared to its control, respectively. (B) Western blotting analysis of expression of IGF/AKT components, including IGF‐I, IGF‐II, IRS‐1, IRS‐2, PIK3CA, AKT, and p‐AKT from indicated cells. Scram, Scramble control; RNAi, SRC‐3–specific shRNA. (C) ChIP analysis of SRC‐3 occupancy on IGF/AKT signaling components in ESCC cell lines. The ChIP assay was done using a‐SRC‐3 antibody. Normal IgG was used as negative control. ESCC, esophageal squamous cell carcinomas.

Downregulation of SRC‐3 is frequently associated with inhibition of IGF/AKT signaling in several human cancers including breast cancer, prostate cancer, and HCC as well 13, 16, 17, 18. To test whether SRC‐3‐mediated ESCC pathogenesis was through IGF/AKT signaling pathway, mRNA and protein levels of numerous well‐known genes of IGF/AKT signaling pathway were compared between control and SRC‐3 knockdown cells by qPCR and Western blot analysis. As expected, parallel to the reduction in SRC‐3, mRNA levels for IGF‐I, IGF‐II, IRS‐1, IRS‐2, PIK3CA, and AKT were all decreased (Fig. 6A). In both Eca109 and EC18 cells, protein level IGF‐1, IGF‐2, and IRS‐1 were dramatically reduced; whereas PIK3CA had moderate reduction and IRS‐2 and p‐AKT only slight reduction in Eca109; PIK3CA and p‐AKT dramatically reduced and IRS‐2 slight reduction in EC18 (Fig. 6B). ChIP assay showed that SRC‐3 was directly recruited onto the promoters of IGF‐I, IGF‐II, IRS‐1, IRS‐2, PIK3CA, and AKT (Fig. 6C), whereas no signals were detected in the negative groups. Additional ChIP primers, primer sets 2, far from start sites of PIK3CA and AKT1 did not detect SRC‐3 binding activity which further demonstrate that the associations was site specific. These results indicated that these genes were direct transcriptional targets of SRC‐3. Overall, these data demonstrated that SRC‐3 indeed regulated the expression of many components of the IGF/AKT pathway in ESCC cell lines.

## Discussion

Previously, our study have showed that SRC‐3 was frequently amplified and overexpressed in ESCC, and the overexpression of SRC‐3 was closely associated with increased cell proliferation and advanced tumor stage 15. However, the role and molecular mechanism by which SRC‐3 regulates ESCC cell aggressiveness remain unclear. In this study, the results of another larger cohort of ESCCs analyzed by IHC validated our previous findings that SRC‐3 was frequently overexpressed in ESCC tissues and it was significantly correlated with advanced tumor stage and seemed to be more common with advanced clinical stage, suggesting that the upregulated expression of SRC‐3 in ESCC may facilitate the invasive phenotype.

Importantly, we further found that patients whose tumor showed SRC‐3 overexpression had inferior survival compared to those with SRC‐3 normal expression, and the overexpression of SRC‐3 was also an independent prognostic predictor in multivariate analysis. This finding in the large‐scale analysis for ESCC patients after curative resection (operable ESCC) is in agreement with previous reports on a small number of patient cases (*n *= 98) with locally advanced disease treated with definite chemoradiotherapy 19. Therefore, SRC‐3 expression could thus be an additional predictive marker for tumor progression for ESCC patients. These findings underscore a potentially important role of SRC‐3 as an underlying biological mechanism in the tumorigenic process of ESCC.

Multiple studies have demonstrated that overexpression of SRC‐3 provides a growth advantage for tumor cells and promotes tumor development through several ways independent of nuclear receptor signaling in various human cancers 6, 13, 14, 20, 21. Cardiac glycoside bufalin, a potent small molecule inhibitor for SRC‐3, has been reported to strongly promote SRC‐3 protein degradation and was able to block cancer cell growth at nanomolar concentrations 22. When incorporated into a nanoparticle delivery system, bufalin was able to reduce tumor growth in a mouse xenograft model of breast cancer. This notion was supported by our findings that shRNA‐mediated SRC‐3 knockdown causes inhibition of cell growth and colony formation in vitro and the in vivo growth inhibition of tumors in nude mice of two different ESCC cell lines.

Malignant tumor cell proliferation is one of the predominant characteristics in cancer development and cell cycle progression is critical for cell proliferation 23. SRC‐3 has been demonstrated to control prostate cancer and other epithelial cancers cell proliferation and tumor growth through modulating cell cycle 8, 13, 24. We observed that reduction in SRC‐3 expression by shRNA in Eca109 and EC18 cells decreased BrdU incorporation and delayed the G1‐S transition. These results suggest that this proliferative effect of SRC‐3 on ESCC cells via promoting DNA synthesis resulting in cell cycle progression from G1 to S phase. Given that SRC‐3 is known to coactivate other transcription factors such as E2F1, AP‐1, Ets‐2, and PEA3 to regulate the expression of cell cycle genes, and these transcription factors have been implicated in ESCC, further work is needed to investigate the potential link between these transcription factors and SRC‐3 and their possible role in the pathogenesis and progression of ESCC 18, 25, 26, 27, 28, 29, 30, 31.

Invasion plays a critical role in cancer progression and is one of the major poor prognostic factors in patients with ESCC. The ability of SRC‐3 promoting cell migration and invasion has been demonstrated in several studies using animal or cell culture models 26, 32, 33. Consistent with these findings, we showed that shRNA‐mediated SRC‐3 knockdown in the Eca109 and EC18 cells inhibited the ability of either cell migration or invasion. Our data indicated that SRC‐3 exerted a vital role to facilitate ESCC cells aggressive phenotype. A limitation of this study should be addressed is that the effect of SRC‐3 on invasion in vivo, in particular on metastasis was not demonstrated. An orthotopic model of human ESCC in nude mouse, simulated human body environment, would better illustrate effect of SRC‐3 on growth and metastasis of tumor in vivo. However, it is really difficult to achieve.

The mechanism of SRC‐3 regulating cancer migration and invasion has been focused on SRC‐3 regulating the expression of matrix metalloproteinases (MMPs) and/or activating focal adhesion kinase (FAK) signaling in previous studies 6, 26, 32, 33, 34. Many studies reported that MMPS and FAK signaling participated in the development of ESCC and resulted in poor prognosis 35, 36, 37. Thus, integrating these studies and our results, it is reasonable to speculate that SRC‐3 may also function through upregulating MMPs expression and activating FAK signaling to enhance human esophageal cancer cell migration and invasion. However, the interaction between SRC‐3 and MMPs and FAK signaling in ESCC need to be further investigated.

**Figure 7:**
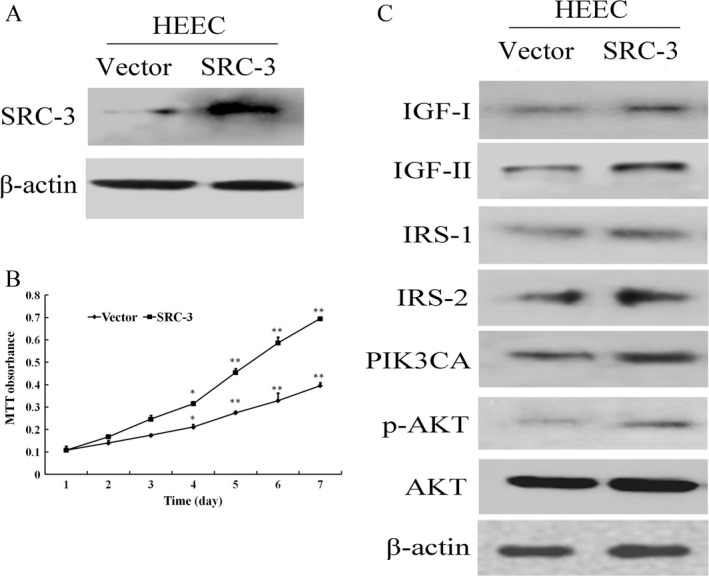
Overexpression of Steroid receptor coactivator (SRC)‐3 promotes human normal esophageal epithelial cell proliferation and activating insulin‐like growth factor (IGF)/AKT signaling pathway. (A) Overexpession of SRC‐3 in immortalized human normal esophageal epithelial cell (HEEC) by immunoblotting. *β*‐actin was used as a loading control. (B) Overexpression of SRC‐3 promotes growth rate of HEEC as determined by MTT assay. (C) Western blotting analysis of expression of IGF/AKT components, including IGF‐I, IGF‐II, IRS‐1, IRS‐2, PIK3CA, AKT, and p‐AKT from indicated cells. *β*‐actin was used as a loading control. MTT, Methyl thiazolyl tetrazolium. *P < 0.05 and **P < 0.01.

IGF/AKT signaling pathway is involved in many important cell processes related to cancer 38, 39. Deregulation of multiple IGF/AKT signaling components have been detected in a wide variety of human carcinomas and considered to play a central role in cancer progression 16, 17, 18, 40, 41. Human esophageal epithelial cells express IGF‐IR, and IGF‐I is known to stimulate both thymidine incorporation and proliferation in these cells 42. Adachi et al. reported that inhibition of IGF‐IR suppressed proliferation, colony formation, and motility through blocking ligand‐induced AKT activation 43. These studies demonstrate that IGF/AKT signaling also play a key role in ESCC cells aggressiveness 42, 43, 44. Accumulated evidences show that SRC‐3 is associated with the regulation of IGF/AKT pathway in several types of cancers. In agreement with these findings, we found that depletion of SRC‐3 also caused a decreased expression of IGF/AKT signaling components both in mRNA and protein level in ESCC cell lines. Moreover, ChIP assay showed that the regulation of these genes by SRC‐3 was at the transcription level and it occurred via direct recruitment of SRC‐3 to the promoters of those genes. In addition, we found that overexpression of SRC‐3 in HEEC not only promoted its growth rates but also enhanced IGF/AKT downstream target genes expression (Fig. 7). Also, the level of SRC‐3 was correlated with p‐AKT expression in our ESCC specimens (data not shown). These suggested that activation of IGF/AKT was also involved in the effect of SRC‐3 on the ESCC cell growth and invasiveness. SRC‐3 has been shown to promote HCC cell proliferation through activation of the AKT signaling pathway to regulate cycle control‐related gene such as inhibit cell cycle inhibitor p21Cip1/Waf1 expression, whereas PI3K/AKT inhibitor LY294002 can abolish the effect of SRC‐3 13. The role of SRC‐3 enhancing HCC cell invasiveness was also demonstrated via PI3K/AKT pathways to upregulating MMP‐9 expression both in vitro and in vivo. Together with these studies, our results support the previous findings that SRC‐3 activating many important components of IGF/AKT pathway at both transcription and phosphorylation levels should lead to tumor progression by ensuring the efficient downstream signaling to enhance growth and invasion 16, 18.

However, SRC‐3 is likely to be involved in multiple signaling pathways during the development of cancer. They are considered as master regulators of differential gene expression and accomplish this through combinatorial codes of posttranslational modifications, which involve mainly phosphorylation, ubiquitination, and sumoylation 22, 27, 45. Future studies are necessary to elucidate detailed mechanisms that affect esophageal cancer behavior.

In summary, our studies illustrate that SRC‐3 overexpression is clinically and functionally relevant to the progression of human ESCC. Understanding the precise role of SRC‐3 in the pathogenesis of ESCC and activation of the IGF/AKT pathway will increase our knowledge of the biologic basis of cancer progression and disruption of functions of SRC‐3 or its target genes and pathways may have potential therapeutic advantages for ESCC.

## Conflicts of Interest

No potential conflicts of interest were disclosed.

## Supporting information

Appendix S1. Supplementary Materials and Methods.
