# Supplementary material for: Overexpression of SRC‐3 promotes esophageal squamous cell carcinoma aggressiveness by enhancing cell growth and invasiveness
Source: Cancer Med. 2016 Oct 26;5(12):3500–11. doi: 10.1002/cam4.884 (PMC5224859; doi:10.1002/cam4.884)
Supplement: Supplementary file 1 — Appendix S1. Supplementary Materials and Methods. [file CAM4-5-3500-s001.pdf]

Overexpression of SRC-3 promotes esophageal squamous cell carcinoma aggressiveness by enhancing cell growth and invasiveness

Fang-Ping Xu, PhD, MD[1]; Yan-Hui Liu, BS[1]; Xin-Lan Luo, BS[1]; Fen Zhang, MS[1]; Hai-Yu Zhou, PhD, MD[2]; Yan Ge, PhD, MD[1]; Chao Liu, PhD, MD[1]; Jie Chen, BS[1]; Dong-Lan Luo, MS[1]; Li-Xu Yan, PhD, MD[1]; Ping Mei, MS[1]; Jie Xu, MS[1] and Heng-Guo Zhuang, MS[1]

[1] *Department of Pathology and Laboratory Medicine, Guangdong General Hospital & Guangdong Academy of Medical Science, Guangzhou, China*

[2] *Department of Thoracic Surgery, Cancer Center, Guangdong General Hospital, Guangzhou, China*

Supplementary Materials and Methods

Immunohistochemistry (IHC)

IHC staining was performed on 4-μm TMA sections rehydrated through gradient alcohols. Endogenous peroxidase activity was blocked with 0.3% hydrogen peroxide for 15 min and antigen were retrieved or 10 min in 10mmol/L citrate buffer (pH 6.0) at 98°C. Nonspecific binding was blocked with 10% normal rabbit serum for 20 min. The TMA slides were incubated with anti-SRC-3 (a monoclonal antibody directed at amino acids 376-389 of SRC-3, Transduction Laboratories, San Jose, CA, 1:50 dilution) for 60 min at 37°C in a moist chamber. Subsequently, the slides were sequentially incubated with biotinylated rabbit antimouse immunoglobulin at a concentration of 1:100 for 30 min at 37°C and then reacted with a streptavidin-peroxdase conjugate for 30 min at 37°C and 3'-3' diaminobenzidine as a chromogen substrate. The nucleus was counterstained using Meyer's hematoxylin. The negative control was performed by replacing the primary antibody with a normal murine IgG. Known immunostaining positive slides were used as positive controls. Positive expression of SRC-3 in ESCC and normal esophageal mucosa cells was primarily a nuclear pattern. The malignant and non-malignant tissues were scored for SRC-3 by assessing the site of positive staining in the nucleus. The staining of the nuclei of the normal esophageal mucosa ranged from 0-10% of epithelium with positive staining, thus, overexpression of SRC-3 was scored when more than 10 percent of tumor cells were positively stained in the nuclei, normal expression level of SRC-3 when less than 10 percent of tumor cells were positive.

Vectors, Retroviral Infection, and stable cell line selection

To silence endogenous SRC-3, the DNA oligonucleotides encoding short hairpin RNA (shRNA, such as 5'-ggatcc ACCCTGAGAGCTTTATTACttcaagaga GTAATAAAGCTCTCAGGGT tttttaagctt- 3', with sequences in capitals targeting human SRC-3 mRNA), were synthesized, annealed, and subcloned into retroviral vector pSuper-retro-puro (Addgene, Cambridge,USA). Inserts were verified by DNA sequencing. For retroviral packaging, constructs and retrovirus Packaging plasmid （PIK） were cotransfected into 293FT cells with Lipofectamine 2000 (Invitrogen) according to the manufacturer's instructions. Virus-containing medium was harvested at 48 and 72 h after transfection and filtered with 0.45-mm MicroFunnel filters (Millipore).

Eca109 and EC18 were infected with culture supernatants from individual 293FT cells at a multiplicity of infection of 10 in the presence of 2ug/mL polybrene (Sigma-Aldrich). Stable cell lines expressing SRC-3 shRNA were selected for 10 days with 0.25μg/mL puromycin 48 h after infection. Following the selection, cell lysates prepared from the pooled population of cells in sampling buffer were fractionated on SDS–PAGE for western blot detection of respective protein levels.

RNA extraction, cDNA synthesis, and qPCR

Total RNA was isolated using TRIzol reagent (Invitrogen) as the manufacturer instructed. Three micrograms of total RNA was used for a reverse transcription

reaction with Moloney murine leukemia virus RT and oligo(dT)18 primers; cDNAs were diluted 10-fold, and a 5mL of dilution was used for the PCRs. Gene sequences were amplified in the presence of SYBR Green fluorophore and detected using ABI Prism 7500 Sequence Detection System (Applied Biosystems). Fluorescent values after each elongation step were collected along with a melting curve analysis at the end of the PCR. Expression data were normalized to the geometric mean of housekeeping gene glyceraldehyde-3-phosphate dehydrogenase (*GAPDH*) to control the variability in expression levels and calculated as $2^{-[(Ct\text{ of gene}) - (Ct\text{ of }GAPDH)]}$, where Ct represents the threshold cycle for each transcript. Primer sequences are listed as below:

SRC-3: forward: 5' TGGAAGACATAAACGCCAGTCC 3'

reverse: 5' TGCAAATCTTCCCCTTCCTCC 3'

IGF1：forward:5' AGGAGGCTGGAGATGTATTG 3'

reverse: 5' GTACTTCCTTCTGGGTCTTG 3'

IGF2：forward: 5' CCGTGCTTCCGGACAACTTC 3'

reverse: 5' CCGATTGCTGGCCATCTCTG 3'

IRS1：forward: 5' GTCAGTAGCTCAACTGGACAT 3'

reverse: 5' CGCTTGGCACAATATAGAACG 3'

IRS2：forward: 5' GCGGCTCGAGTACTACGAGA 3'

reverse: 5' CGGCGAAGTACTCGTCCTTG 3'

PIK3CA：forward: 5' AACCTCAGGCTTGAAGAGTG 3'

reverse: 5' GTCACCGATTGACAGACAAC 3'

AKT：forward: 5' AGCACCGCGTGACCATGAAC 3'

reverse: 5' CGTCCTTGGCCACGATGACT 3'

Western Blot Analysis

Cells were rinsed twice with phosphate buffered saline (PBS) buffer, and cell pellets were frozen at -80°C until use. Treated cells were lysed in lysis buffer (20 mM Tris-Cl [pH 8.0], 125 mM NaCl, 1% Triton X-100, 2 mM EDTA, 0.2 mM NaF, 100 μg of PMSF/mL, protease inhibitor cocktail) for 30 min on ice with constant vigorous vortexing. The debris was cleared by centrifugation at 12,000 rpm for 10 min at 4°C. Lysates were boiled in gel loading buffer and separated on 5% to 8% sodium dodecyl sulfate–polyacrylamide (SDS-PAGE) gradient gels and transferred to nitrocellulose membranes. After blocked with 5% non-fat milk in PBS with 0.2% Tween-20, membranes were incubated overnight at 4°C with primary antibodies listed as below, followed by horseradish peroxidase-conjugated secondary antibodies for 1 h at room temperature. All blots were developed with enhanced chemiluminescence Western blotting detection reagent (Amersham Biosciences). Signal intensities were determined by densitometry and normalized using anti-β-actin antibodies.

Primary antibodies and titers for Western blotting:

SRC-3, SRC-3 (5E11) Rabbit mAb, Cell Signaling, #2126, 1:1000

β-actin, β-actin antibody (AC-74), Mouse, Sigma-Aldrich, Cat# A5316, 1:3000

IGF-I, IGF-I Antibody, Rabbit, Boster, BA0498, 1:200

IGF-II, IGF-II Antibody, Rabbit, Boster, BA0942, 1:200

IRS-1, IRS-1 (59G8) Rabbit mAb, Cell Signaling, #2390, 1:1000

IRS-2, IRS-2 (L1326) Antibody, Rabbit, Cell Signaling, #3089, 1:1000

PIK3CA, PI3 Kinase p110α (C73F8) Rabbit mAb, Cell Signaling, #4249, 1:1000

AKT, Akt (pan) (C67E7) Rabbit mAb, Cell Signaling, #4691, 1:1000

p-AKT, Phospho-Akt (Ser473) Antibody, Rabbit ,Cell Signaling, #9271, 1:1000

5-bromo-2'-deoxyuridine (BrdU) incorporation assays

For BrdU incorporation, cells were seeded on poly-L-lysine coated glass coverslips in six-well plates and BrdU was added to the culture medium 1h before fixation. The cells were fixed in 95% methanol at 4°C, denatured in 2 mol/L of HCl and stained with mouse anti–BrdU fluorescein conjugate (Roche, Basel, Switzerland), according to the manufacturer's recommendations. DAPI was used as the nuclear counterstain. Microscopic fields were selected randomly and ≥200 cells were counted under each condition. BrdU-positive cells were calculated as the percentage of total cells in each field.

Methyl thiazolyl tetrazolium (MTT) assays

Cell viability assays and growth curve analysis were evaluated by MTT assay as described elsewhere. Briefly, a total of $1x10^3$ cells were seeded in 96-well plates and MTT (5 mg/mL) was added to each well every 24 h. The plates were incubated at

37°C for 4 h, and then 100μL dimethylsulfoxide (DMSO) were added to each well to lyse the cells. The absorbance at 490 nm was then recorded by a microplate reader (Bio-Tek).

Colony formation and Soft argar assays

Cells were collected with trypsin and seeded into 100-mm dishes at a concentration of 1 $\times 10^3$ after counting. The plates were incubated at 37°C in a humidified incubator containing 5% CO_2. When formation of colonies was visible (2-3 weeks), they were fixed with 4% formaldehyde, stained with crystal violet, and counted.

A soft agar colony formation assay was used to assess the anchorage-independent growth ability of cells. Cells were resuspended in 0.33% soft agar with 1 mL of growth medium (Dulbecco's modified Eagle medium supplemented with 10% fetal bovine serum) and layered onto 0.66% solidified agar in six well plates. The soft agar colonies were allowed to grow for two weeks at 37°C. Colonies greater than 100 μm in diameter at low magnification (×100) were scored as positive and counted at four points on each well.

Flow cytometry assay

Flow cytometry assay was done by propidium iodide staining. Cells were grown to 70% to 80% confluence, then harvested and fixed overnight at 4°C in 70% ethanol. After washed with PBS, cells were incubated with 5μg/μL propidium iodide and

50μg/μL RNase in PBS for 30 min at 37°C. Each sample was analyzed by fluorescence-activated cell sorter analysis (FACS) (BD, San Jose, CA, USA). The cell cycle distribution was established by plotting the intensity of the propidium iodide signal, which reflects the cellular DNA content.

Wound healing assay

Cell migration was assessed by measuring the movement of cells into a scraped, acellular area created by a 10μl pipette tip, and the spread of wound closure was observed after and photographed at 0, 10 and 20 h under a microscope.

Transwell assay

Invasion assays were performed in 24-well Bio-Coat Matrigel Invasion Chambers (BD Biosciences) according to the manufacturer's instructions. In brief, 2×10^4 cells were added to the upper chamber, and 10% fetal bovine serum in Dulbecco's modified Eagle medium was added to the bottom chamber as a chemoattractant. After 24 h of incubation at 37°C, the non-migrating cells on the upper chambers were removed by a cotton swab, and cells invaded through the matrigel layer to the underside of the membrane were stained with hematoxylin, and counted (10 random 100×fields per well).

Chromatin Immunoprecipitation (ChIP) assay

ChIP assay were performed using a Magna ChIP Assay Kit (Millipore, Billerica,

MA) according to the manufacturer's protocol. In briefly, cells were treated with 1% formaldehyde for 10 min to cross-link associated protein to DNA, lysed, and then sonicated. The crude chromatin solution was diluted and incubated at 4°C with specific antibodies overnight, or negative control mouse immunoglobulin G. PCR was performed using 5 μL of purified ChIP DNA for 28 cycles with promoter-specific primers. Primer sequences are listed as below:

IGFI: forward: 5' TTGTCACCATGCCCAAAAAA 3'

□ reverse: 5' TTGCGCAGGCTCTATCTGC 3'

IGFII: forward: 5' GCCGCCTCCTCTTCATCTAC 3'

reverse: 5'CGGGCGCCCAGCTCGGTTTG 3'□

IRS1: forward: 5'AGCTGGCACCATCCTTGTT 3'□

reverse: 5'□CTTGCCCATCCGTGTGAC 3'□

IRS2: forward: 5'GCCGGCATCCACAACAA 3'□

reverse: 5'CTAAGAAGAGCAAAACAACA 3'□

primer 1 sets of PIK3CA: forward:5'GTTGAGGGCAGGGAGTGAA 3'

reverse: 5'GAGGCTATGGGAAGAATGAA 3'□

primer 2 sets of PIK3CA: forward: 5' GCCTTTTGATATACTCTTACA 3'□

reverse: 5'TCTGACTGAAATGAATCTACA 3'□

primer 1 sets of AKT1: forward: 5' GGGGCGCTGTGGTTTAGG 3'□

reverse: 5'ATGGCCCCGTTTGCTCTC3'□

primer 2 sets of AKT1: forward: 5' CCAGGTGGCCACTTCTTGA 3'□

reverse: 5'TGCCGCCTGCCTTTACCAT 3'

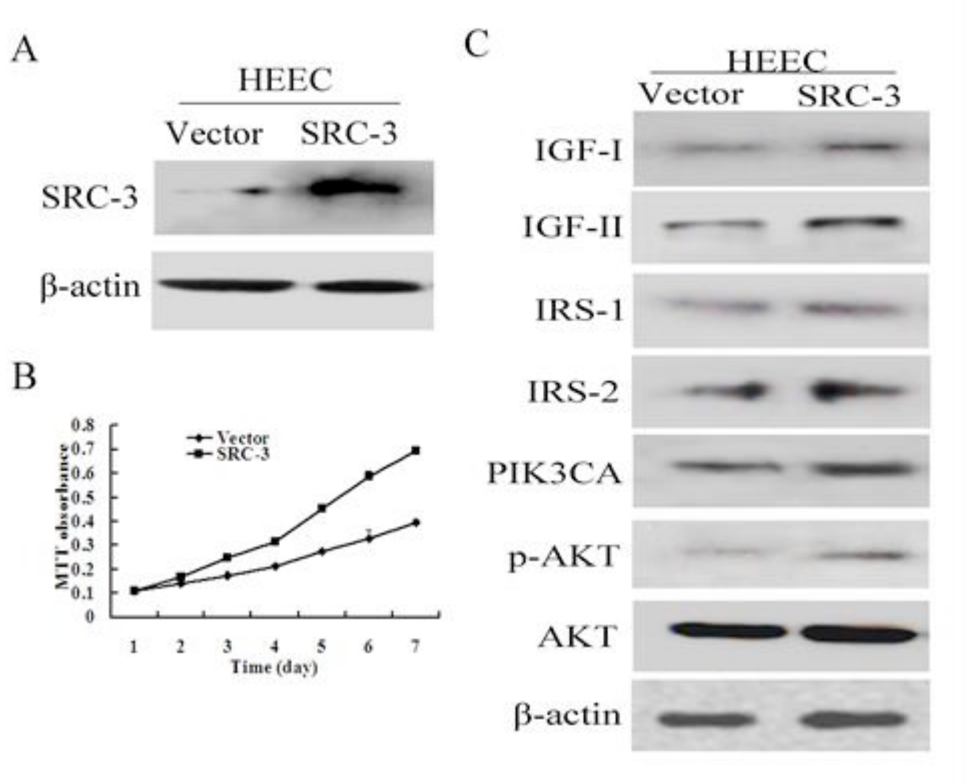

Supplementary Figure 1. Overexpression of SRC-3 promotes human normal esophageal epithelial cell proliferation and activating IGF/AKT signaling pathway. (A) Overexpession of SRC-3 in immortalized human normal esophageal epithelial cell (HEEC) by immunobloting. β-actin was used as a loading control. (B) Overexperssion of SRC-3 promotes growth rate of HEEC as determined by MTT assay. (C) Western blotting analysis of expression of IGF/AKT components, including IGF-I, IGF-II, IRS-1, IRS-2, PIK3CA, AKT and p-AKT from indicated cells. β-actin was used as a loading control.
